# The expression and significance of long non-coding RNA ITGB2-AS1 in renal clear cell carcinoma

**DOI:** 10.15537/smj.2023.44.1.20220533

**Published:** 2023-01

**Authors:** Wei Zhou, Zhi-Gang Liu, Lian-Qu Wang

**Affiliations:** *From the School of Nursing and Health (Zhou), Henan University, from the Department of Orthopedic (Liu), and from the Department of Urinary Surgery (Wang), The First Affiliated Hospital of Henan University, Henan, China.*

**Keywords:** kidney renal clear cell carcinoma, long non-coding RNA, ITGB2-AS1, miR-338-3p, EGFR

## Abstract

**Objectives:**

To explore the expression and significance of long non-coding RNA ITGB2-AS1 in kidney renal clear cell carcinoma (KIRC).

**Methods:**

The expression of ITGB2-AS1 in KIRC tissues of 45 KIRC patients in the first affiliated hospital of Henan University, Henan, China, from September 2018 to December 2020, KIRC cells were detected and the relationship of ITGB2-AS1 and overall survival of KIRC patients were analyzed. The expression of ITGB2-AS1 in KIRC cells Caki-1 and ACHN was interfered, and the changes of cell proliferation, invasion, migration, and apoptosis were detected. Dual luciferase reporter gene assay and RNA pull-down assay were carried out to verify the relationship between ITGB2-AS1 and miR-338-3p or miR-338-3p and epidermal growth factor receptor (EGFR). The expression of miR-338-3p and EGFR were detected after the interference of ITGB2-AS1.

**Results:**

The expression of ITGB2-AS1 was expressed highly in KIRC tissues and cells (*p*<0.05). The overall survival of KIRC patients with high ITGB2-AS1 was poorer than those with low ITGB2-AS1. In Caki-1 cell, downregulation of ITGB2-AS1 suppressed the cell proliferation, invasion and migration, promoted the cell apoptosis (*p*<0.05). In ACHN cell, upregulation of ITGB2-AS1 promoted the cell proliferation, invasion and migration and inhibited the apoptosis (*p*<0.05). The ITGB2-AS1 targeted and regulated the expression of miR-338-3p/EGFR.

**Conclusion:**

The ITGB2-AS1 is expressed highly in KIRC and affects the survival of patients by regulating cell proliferation, invasion, and apoptosis.

**K**idney cancer is the second largest malignant tumor in the urinary system after bladder cancer.^1^ Kidney renal clear cell carcinoma (KIRC) is the most common pathological type of renal carcinoma, with the higher morbidity and mortality, and has the highest invasive and metastatic potential, leading to a 5-survival rate less than 10%.^2,3^ Therefore, it is of great significance for the clinical diagnosis and therapy for KIRC patients to explore a new molecular biomarker and in-depth understanding pathogenesis of KIRC.

Multiple long non-coding RNA (lncRNA) had been verified to be involved in the process and prognosis of various cancer types such as breast cancer, colorectal cancer, and KIRC.^4-6^ As a newly identified lncRNA, ITGB2-AS1 had been discovered to be abnormally expressed in breast cancer and osteosarcoma, and it could regulate the proliferation, migration and invasion of various cancer cells.^7,8^ However, there are few studies on the expression significance and biological functions of ITGB2-AS1 in KIRC. The role and molecular mechanism of ITGB2-AS1 in KIRC still need to be further investigated.

In the present study, the expression significance of ITGB2-AS1 in KIRC tissues were investigated clinically and through the gene expression profiling interactive analysis (GEPIA) database, and the effect of ITGB2-AS1 on the biological behavior of KIRC cells were explored in the cell experiments. The aim of this study was to reveal the significance and mechanism of ITGB2-AS1 in KIRC, and to provide new target for the diagnosis and treatment of renal cancer.

## Methods

A total of 45 KIRC tissues and para-cancerous tissue specimens (>2 cm) were collected from KIRC patients who underwent surgical resection in the first affiliated hospital of Henan University, Henan, China, from September 2018 to December 2020. Tissue specimens were removed and placed in liquid nitrogen and stored at -80°C. Inclusion criteria included: I) diagnosed as KIRC pathologically and underwent surgical resection; II) did not receive any chemotherapy or radiotherapy before surgery; and III) completed clinical data. Exclusion criteria was any patient with autoimmune diseases and family hereditary history. Among the 45 patients, 30 were male and 15 were female (45-80 years old), mean age was 63.91±6.94 years old. Tumor node metastasis (TNM) stage included: 27 cases with I-II, 18 cases with III-IV; and 17 cases with lymph node metastasis. The follow-up was completed in March 2022, and the follow-up time was 9-42 months. The study had been approved by the ethics committee of the first affiliated hospital of Henan University, Henan, China, and the study procedures followed were in accordance with the principles of Helsinki Declaration of 1975, as revised in 2000.

Human kidney-2 (HK-2) cells (Shanghai Cell Bank, Chinese Academy of Sciences, China) were cultured in DMEM/F12 medium (Solabio, Beijing, China) containing 10% of fetal bovine serum (FBS); A498, 786-O, ACHN, and Caki-1 cells (Shanghai Cell Bank, Chinese Academy of Sciences, China) were cultured in RPMI1640 with 10% FBS. All cells were routinely cultured in 37°C, saturated humidity, and 5% CO_2_.

Total RNA from tissue samples and cells were extracted by Trizol. The concentration and purity of RNA were determined by ultraviolet absorption method. Single-stranded cDNA was synthesized by RNA reverse transcription, and real-time fluorescence quantitative PCR (qRT-PCR) amplification was carried out according to the SYBR Premix Ex Taq II manual with the cDNA as a template. The amplification condition was as follow: 95°C 60 seconds (s), 40 cycles; 95°C 15 s, 58°C 30 s, 72°C 30 s. Primers (Sangon Biotechnology, Shanghai, China) were as follow: ITGB2-AS1 F: 5’-AGGAGATGGAACGAGGAAA-3’, R: 5’-AGTCTTCTGGGTGGCAGTGAT-3’; miR-338-3p F: 5’-GGGTCCAGCATCAGTGATT-3’, R: 5’-GTGCAGGGTCCGAGGT-3’; U6 F: 5’-CTCGCTTCGGCAGCACA-3’, R: 5’-AACGCTTCACGAATTTGCGT-3’; β-actin F: AATCGTGCGTGACATTAAGGAG, R: ACTGTGTTGGCGTACAGGTCTT.

The levels of ITGB2-AS1 and miR-338-3p mRNA were calculated by 2^-ΔΔ^Ct values with U6 and β-actin as internal.

### Transfection

The si-ITGB2-AS1 and siRNA-NC were synthesized by Jima Pharmaceutical Technology (Shanghai, China). The empty vector plasmid pcDNA3.1 were purchased from Jima Pharmaceutical Technology (Shanghai, China), to construct the pcDNA3.1-ITGB2-AS1 overexpression vector plasmid. Then the Caki-1 cell were transfected with siRNA-ITGB2-AS1 and siRNA-NC, and the ACHN cell were transfected with pcDNA3.1-ITGB2-AS1 overexpression vector plasmid and the empty vector plasmid pcDNA3.1, as follow: The cell at logarithmic growth stage were inoculated into a 6-well plate and conventionally cultured, when the fusion degree reached 75%, transfection was carried out according to the instructions of transfection reagent Lipofectamine RNAiMAX or Lipofectamine® 3000 (Thermo fisher, USA). After transfection for 48 hours, the expression of ITGB2-AS1 was detected by qRT-PCR to evaluate the transfection effect.

### MTT assay

Caki-1 and ACHN cells in each group were collected after transfection for 48 hours and inoculated into a 96-well plates with 2×10^5/well; after cultured for 24 hours, 48 hours, and 72 hours, 20 µL MTT reagent (concentration 5 g/L, Amyjet Scientific, Wuhan, China) was added to each well. After incubated for 4 hours, 150 µL dimethyl sulfoxide (DMSO, Solarbio, Beijing, China) was added and shaked for 15 minutes, then the optical density value at 450nm of each well was detected by microplate reader.

### Transwell assay

After transfection for 48 hours, the Caki-1 cell and ACHN cell were cultured with the fresh serum-free RPMI1640 medium. A 100 µL cell suspension (1×10^5 cells) were added into the upper chamber coated with Matrigel (Corning, USA); then, 600 µL medium containing 10% FBS were added into the lower chamber. After cultured for 24 hours, the cells in the upper chamber were wiped off and the cells in the lower chamber were fixed with methanol for 30 minutes and stained by 0.1% crystal violet for 10 minutes. After washing the dye, the number of invaded cells were observed under an inverted microscope (200×) to evaluate the cell invasion ability.

### Scratch assay

After transfection for 48 hours, the Caki-1 cell and ACHN cell at logarithmic growth stage were digested by ethylene diamine tetraacetic aicd (EDTA) and adjusted cell concentration at 1×10^6 /mL. A 1 mL cell was seeded into a 6-well plates. When cells reached 90% confluence, a wound was scratched by utilizing with a 200 µL micropipette tip. After washing with phosphate buffer solution (PBS), the cells were cultured in the serum-free medium for 48 hours. The inverted microscope was used to photograph at 0 hour and 48 hours. The healing rate of scratches was as follow: 0 hour scratch width-48 hours scratch width)/0 hour scratch width×100%.

### Flow cytometry

The Caki-1 and ACHN cells after transfection for 48 hours were collected, they were resuspended by pre-cooled PBS and centrifuged at 1000 rpm for 5 minutes. The binding buffer was added to adjust the cell concentration to 1×10^6 cells/mL. A 5 µL AnnexinV-APC was added in 100 µL cell suspension and incubated for 5 minutes at room temperature in the dark and 10 µL 7-AAD were added according to the manufacture’s protocol. Flow cytometry (FCM) analysis was used to detect the cell apoptosis rate of Caki-1 cell and ACHN cell.

### Double luciferase reporter gene assays

The online software: LncBase Predicted v.2 and Starbase 3.0 were used to predict the binding sites of miR-338-3p with ITGB2-AS1 and 3’untranslated regions (UTR) of epidermal growth factor receptor (EGFR) mRNA. The sequence fragments containing the wide type (WT) or mutation (MUT) binding sites of miR-338-3p with ITGB2-AS1 and EGFR were amplied and cloned into the pmiRGLO vector to construct the double luciferase reporter vectors. The ITGB2-AS1-WT, ITGB2-AS1-MUT, EGFR-WT, and EGFR-MUT vectors were co-transfected with miR-338-3p mimic and its negative control (Ruibo BioTech, Guangzhou, China) into 293T cell. All steps were referred to the Lipofectamine® 3000 protocols. After transfection for 48 hours, the relative luciferase activity was detected according to the instructions of the dual-luciferase reporter assay kit (Beyotime, Shanghai, China).

### Pull down assay

Biotinylated miR-338-3p and the corresponding mutant or biotinylated negative control were synthesized and purchased from RiboBio (Guangzhou, China). The oligonucleotides were transfected into Caki-1 and ACHN cells using Lipofectamine 3000 (Invitrogen, USA). After incubation for 48 hours, the cells were lysed by lysis buffer and incubated with Dynabeads M-280 Streptavidin (Invitrogen, USA), according to the manufacturer’s protocols. Afterwards, the beads were washed with pre-cooled lysis buffer, the RNA complexes bound to the beads were eluted and purified using Trizol. The expression of ITGB2-AS1 and EGFR were detected by qRT-PCR.

### Western blot

The protein was extracted from cells by RIPA lysate, and the protein concentration was determined by BCA protein assay kit (Beyotime, Shanghai, China). Then, sodium dodecyl sulfate polyacrylamide gel electrophoresis (SDS-PAGE) was carried out to separate the protein and the separated protein was transformed into the polyvinylidene fluoride (PVDF) membrane. Mouse anti-human EGFR (1:10 000, Proteintech, Wuhan Sanying, China) and GAPDH (1:2000, Proteintech, Wuhan Sanying, China) primary antibodies were incubated at 4°C overnight. After washing, horseradish peroxidase labeled sheep anti-mouse secondary antibody (Proteintech, Wuhan Sanying, China) was incubated at room temperature for one hour. Electrochemical luminescence developer was added, and the relative protein level was calculated using GAPDH as internal reference.

### Statistical analysis

Measurement data were represented by mean and standard deviation (SD), and the Statistical Package for the Social Sciences, version 25.0 (IBM Corp., Armonk, NY, USA) was used to carry out statistical analysis. Independent sample T-test was used for comparison between the 2 groups, and one-way analysis of variance was used for comparison between multiple groups. The student-Newman-Keuls (SNK-q) test was used for further pairwise comparisons between 2 groups, and the χ^2^ test was used to compare the enumeration data between groups. A *p*-value of <0.05 was considered significant.

## Results

**Figure 1:**
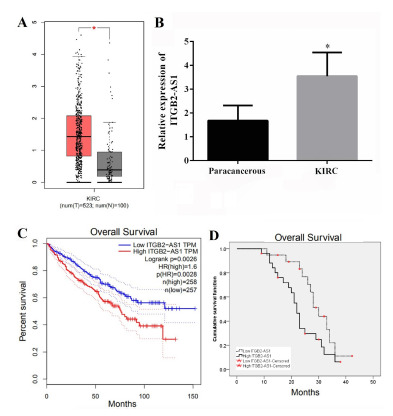
- The expression of ITGB2-AS1 in kidney renal clear cell carcinoma (KIRC) and correlation with prognosis. **A**) The expression of ITGB2-AS1 in KIRC analyzed by gene expression profiling interactive analysis (GEPIA) database. **B**) The expression of ITGB2-AS1 in KIRC and adjacent tissues from 45 patients with KIRC was detected by real-time quantitative polymerase chain reaction. **C**) Survival analysis results of 515 KIRC patients retrieved from GEPIA database. **D**) The relationship between ITGB2-AS1 expression and overall survival of 45 KIRC patients was analyzed by Kaplan-Meier. ^*^ *P*-value of <0.05.

Kidney renal clear cell carcinoma data set in the GEPIA database was used to analyze the correlation between ITGB2-AS1 and KIRC. The results showed that the expression of ITGB2-AS1 in KIRC tissues from 523 cases were significantly higher than that in adjacent tissues from 100 cases (*p*<0.05, Figure 1A). The results of qRT-PCR also showed that the expression of ITGB2-AS1 in KIRC tissues was significantly higher than that in adjacent tissues (*p*<0.05, Figure 1B).

A total of 45 KIRC patients were divided into the high-expression group (n=26) and the low-expression group (n=19) according to the mean value of ITGB2-AS1 expression. There were differences in TNM stage and Fuhrman class between the 2 groups (*p*<0.05, Table 1). The results of GEPIA database analysis found that the ITGB2-AS1 high expression group had a lower overall survival of KIRC patients compared with the low expression group (*p*<0.05, Figure 1C). The results of Kaplan-Meier curve of 45 KIRC patients also showed that the overall survival of the high expression group was lower than that of the low expression group (χ^2^=4.571, *p*=0.033; Figure 1D).

**Table 1:** - Relationship between ITGB2-AS1 expression and clinicopathological characteristics of kidney renal clear cell carcinoma patients.

Variables	n	ITGB2-AS1		Comparison
		Low expression group (n=19)	High expression group (n=26)	
***Gender***				
Male	27	11 (57.9)	16 (61.5)	χ^2^=0.184
Female	18	8 (42.1)	10 (38.5)	*p*=0.668
***Age***				
<65	25	11 (57.9)	14 (53.8)	χ^2^=0.073
≥65	20	8 (42.1)	12 (46.1)	*p*=0.787
***Smoking history***				
No	29	13 (68.4)	16 (61.5)	χ^2^==0.227
Yes	16	6 (31.6)	10 (38.5)	*p*=0.634
***Tumor diameter (cm)***				
<5	27	9 (47.4)	18 (66.7)	χ^2^==2.186
≥5	18	10 (52.6)	8 (44.4)	*p*=0.239
***TNM stage***				
I/II	27	15 (78.9)	12 (46.1)	χ^2^==4.919
III/IV	18	4 (21.0)	14 (53.8)	*p*=0.035
***Fuhrman class***				
I/II	24	14 (73.7)	10 (38.5)	χ^2^==6.910
III/IV	21	5 (26.3)	16 (61.5)	*p*=0.009
***Lymph node metastasis***				
No	28	15 (78.9)	13 (50.0)	χ^2^==3.913
Yes	17	4 (21.0)	13 (50.0)	*p*=0.065

Values are presented as numbers and precentages (%). TNM: tumor node metastasis

**Figure 2:**
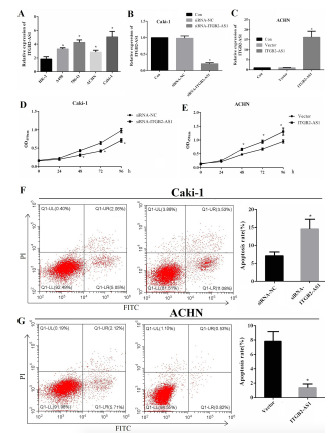
- The effect of ITGB2-AS1 on the proliferation and apoptosis of kidney renal clear cell carcinoma (KIRC) cells. **A**) The expression of ITGB2-AS1 in human kidney-2 (HK-2) and KIRC cell lines (A498, 786-O, ACHN, and Caki-1), ^*^ *p*<0.05, versus HK-2 cell. **B**) The expression of ITGB2-AS1 in Caki-1 cell after transfected with siRNA-ITGB2-AS1, ^*^ *p*<0.05, versus the siRNA-NC group. **C**) The expression of ITGB2-AS1 in ACHN cell after transfected with pcDNA3.1-ITGB2-AS1, ^*^ *p*<0.05, versus the siRNA-NC group. **D**) The proliferation activity of Caki-1 cell after down-regulation of ITGB2-AS1. **E**) The proliferation activity of ACHN cell after up-regulation of ITGB2-AS1, ^*^ *p*<0.05, versus the Vector group. **F**) The apoptosis rate of Caki-1 cell after down-regulation of ITGB2-AS1, ^*^ *p*<0.05, versus the siRNA-NC group. **G**) The apoposis rate of ACHN cell after up-regulation of ITGB2-AS1, ^*^ *p*<0.05, versus the Vector group.

The expression level of ITGB2-AS1 in A498, 786-O, ACHN, and Caki-1 cells were all significantly higher than HK-2 cell (*p*<0.05), and the expression was highest in Caki-1 cell and lowest in ACHN cell (Figure 2A). The expression of ITGB2-AS1 in the siRNA-ITGB2-AS1 group was significantly lower than that in the siRNA-NC group and Con group (*p*<0.05, Figure 2B), and the expression of ITGB2-AS1 in the ITGB2-AS1 group was significantly higher than that in Vector group and Con group (*p*<0.05, Figure 2C). The results of the MTT assay showed that the proliferation of Caki-1 cell in the siRNA-ITGB2-AS1 group was significantly reduced, compared with the siRNA-NC group (*p*<0.05, Figure 2D) and the proliferation of ACHN cell in the ITGB2-AS1 group was significantly increased, compared with the Vector group (*p*<0.05, Figure 2E).

The apoptosis rate of Caki-1 cell in the siRNA-ITGB2-AS1 group was significantly increased, compared with the siRNA-NC group (*p*<0.05, Figure 2F), and the apoptosis rate of ACHN cell in the ITGB2-AS1 group was significantly lower than that in the Vector group (*p*<0.05, Figure 2G).

**Figure 3:**
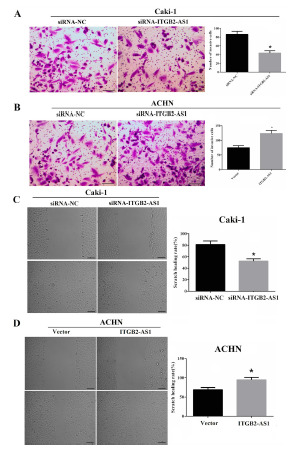
- The effect of ITGB2-AS1 on invasion and migration abilities of kidney renal clear cell carcinoma (KIRC) cells. **A**) The invasion ability of Caki-1 cell after down-regulation of ITGB2-AS1, scale bar: 50 µm, ^*^ *p*<0.05, versus the siRNA-NC group. **B**) The invasion ability of ACHN cell after up-regulation of ITGB2-AS1, scale bar: 50 µm, ^*^ *p*<0.05, versus the Vector group. **C**) The migration ability of Caki-1 cell after down-regulation of ITGB2-AS1, scale bar: 100 µm, ^*^ *p*<0.05, versus the siRNA-NC group. **D**) The migration ability of ACHN cell after up-regulation of ITGB2-AS1, scale bar: 100 µm, ^*^ *p*<0.05, versus the Vector group.

Transwell assay showed that the invasion numbers of Caki-1 cell in the siRNA-ITGB2-AS1 group were significantly reduced, compared with the siRNA-NC group (*p*<0.05, Figure 3A); and compared with the Vector group, the invasion numbers of ACHN cell in the ITGB2-AS1 group were significantly increased (*p*<0.05, Figure 3B).

The scratch healing rate of Caki-1 cell in the siRNA-ITGB2-AS1 group was significantly reduced, compared with the siRNA-NC group (*p*<0.05, Figure 3C) and the scratch healing rate of ACHN cell in ITGB2-AS1 group was significantly higher than that in Vector group (*p*<0.05, Figure 3D).

**Figure 4:**
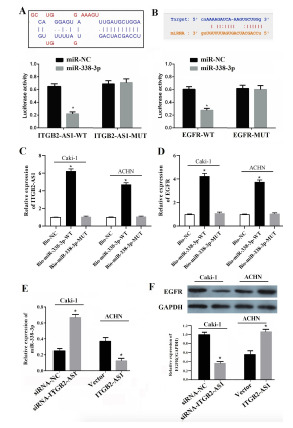
- ITGB2-AS1 targeted regulate miR-338-3p/epidermal growth factor receptor (EGFR) axis in kidney renal clear cell carcinoma (KIRC). **A**) The binding site of miR338-3p in the ITGB2-AS1 was predicted by LncBase Predicted v.2 and verified by dual luciferase reporter gene assay, ^*^ *p*<0.05, versus the miR-NC group. **B**) The binding site of miR-338-3p in 3’UTR of EGFR was predicted by Starbase 3.0 and verified by dual luciferase reporter gene assay, ^*^ *p*<0.05, versus the miR-NC group. **C&D**) The direct correlation between ITGB2-AS1 and miR-338-3p or miR-338-3p and EGFR, both in Caki and ACHN cells, were confirmed by RNA pull-down assay, ^*^ *p*<0.05, versus the Bio-NC group. **E**) The expression of miR-338-3p both in Caki and ACHN cells after down-regulation or up-regulation of ITGB2-AS1, ^*^ *p*<0.05, versus the siRNA-NC or Vector groups. **F**) The expression of EGFR protein both in Caki and ACHN cells after down-regulation or up-regulation of ITGB2-AS1, ^*^ *p*<0.05, versus the siRNA-NC or Vector groups.

LncBase Predicted v.2 and Starbase 3.0 predicted that there was complementary binding site between miR-338-3p and ITGB2-AS1, miR-338-3p, and EGFR (Figure 4A&B). Then the dual luciferase reporter gene assay was carried out to verify the targeted relationship. The results showed that miR-338-3p mimics can reduce the luciferase activity of 293T cell transfected with ITGB2-AS1-WT vector or EGFR-WT (*p*<0.05, Figure 4A&B). As presented in the Figure 4C&D, RNA pull down assay also showed that compared with Bio-NC, treatment with Bio-miR-338-3p WT significantly increased the enrichment of ITGB2-AS1 and EGFR (*p*<0.05), both in Caki-1 and ANCH cells, while no significant difference was found in the enrichment of ITGB2-AS1 and EGFR following treatment with Bio-miR-338-3p MUT (*p*>0.05).

Then qRT-PCR and Western blot were carried out to detect the expression of miR-338-3p or EGFR after downregulation or upregulation of ITGB2-AS1, the results showed that the expression of miR-338-3p in siRNA-ITGB2-AS1 group was significantly increased compared with the siRNA-NC group, while in the ITGB2-AS1 group was significantly decreased compared with the Vector group (*p*<0.05, Figure 4E). The expression of EGFR in siRNA-ITGB2-AS1 group was significantly decreased compared with the siRNA-NC group, while in the ITGB2-AS1 group was significantly increased compared with the Vector group (*p*<0.05, Figure 4F).

## Discussion

Long non-coding RNA is a kind of long chain RNA without protein coding function, and plays an important regulatory role in gene transcription and chromosome remodeling.^9,10^ A large number of studies have confirmed that a variety of lncRNA were abnormally expressed in KIRC, such as SNHG311 and Fer1L412 were highly expressed in KIRC, while PVT113 and HOTAIRM114 were down-regulated in the KIRC and in the KIRC datasets from The Cancer Genome Atlas (TCGA) database, there were many lncRNAs that were related to the prognosis of KIRC, maybe potential targets for predicting KIRC prognosis.^15,16^ At present, there are few studies on ITGB2-AS1 in KIRC. In the latest study, ITGB2-AS1 had been confirmed higher expression in KIRC tissues and associated with prognosis with clear cell renal cell carcinoma.^17^ In this study, the results from GEPIA database and clinical data showed that the expression of ITGB2-AS1 in KIRC tissues was significantly higher than that in the adjacent tissues and the higher expression of ITGB2-AS1 was closely related to the poor prognosis of KIRC patients. These results suggested that ITGB2-AS1 might play an important regulatory role in the occurrence and development of KIRC. Some studies have confirmed that ITGB2-AS1 was closely related to tumor occurrence and development. For example, Liu et al^8^ pointed out that ITGB2-AS1 overexpression could promote the migration and invasion of breast cancer cells by up-regulating ITGB2 expression. Dai et al^7^ found that knockout of ITGB2-AS1 expression could inhibit proliferation, invasion and migration of osteosarcoma cells by inhibiting Wnt/β-catenin pathway. In this study, down-regulation of ITGB2-AS1 inhibited the proliferation, invasion and migration of Caki-1 cell, while the up-regulation of ITGB2-AS1 promoted the proliferation, invasion and migration of ACHN cell. The results suggest that ITGB2-AS1 highly expressed in KIRC may play an important role in carcinogenesis by regulating cell proliferation, invasion and migration.

In general, the lncRNA can competitively combine with miRNA to play the role of a sponge, thus influencing downstream mRNA to play a variety of biological effects.^18,19^ For example, silencing SBF2-AS1 exerted suppressive effects on KIRC by elevating miR-338-3p and suppressing ETS1 and Circ_0000274 RNA functioned as an oncogene in RCC development by regulating miR-338-3p RNA/NUCB2 protein axis.^20,21^ The existing study had confirmed that miR-338-3p was down-regulated in KIRC cells, and inhibit KIRC cell proliferation, migration and invasion by targeting different mRNA.^20,22^ As for ITGB2-AS1, Zhang et al^17^ had verified that silencing ITGB2-AS1 inhibited the cell proliferation, promoted apoptosis in ccRCC cells by modulating miR-328-5p/HMGA1 axis. In order to explore the potential mechanism of ITGB2-AS1 in KIRC, the bioinformatics tools LncBase Predicted v.2 or Starbase predicted that there was potential target site of miR-338-3p on ITGB2-AS1 and 3’UTR of EGFR, suggesting that miR-338-3p was a target gene of ITBG2-AS1, and EGFR was a target gene of miR-338-3p. Double luciferase reporter gene assay and RNA pull down assay had been confirmed that ITGB2-AS1 could directly interact with miR-338-3p, and miR-338-3p could directly interact with EGFR. In addition, after down-regulating ITGB2-AS1 in Caki-1 cell, the miR-338-3p was up-regulated and the expression of EGFR was down-regulated and after up-regulating ITGB2-AS1 in ACHN cell, the miR-338-3p was down-regulated and the expression of EGFR was up-regulated. It was speculated that ITGB2-AS1 may targeted regulate miR-338-3p/EGFR axis in KIRC cells, and the sponge function of ITGB2-AS1 between miR-338-3p and EGFR might be one of the molecular mechanisms of ITGB2-AS1 involved in the development of KIRC. However, to fully confirm the mechanism, rescue experiments with miR-338 up-regulators and EGFR inhibitors still need to be carried out in further experiments.

### Study limitations

In this study, due to the limitation of funds and time, the rescue experiments were not carried out, but in further studies, it will be discussed in depth.

In conclusion, ITGB2-AS1 was highly expressed in KIRC and closely related to the prognosis of KIRC. The ITGB2-AS1 played a role in promoting cell proliferation, invasion and migration in KIRC, and its molecular mechanism may be related to the targeted regulation of miR-338-3p. The results in this study revealed that the expression significance and mechanism of ITGB2-AS1 in KIRC preliminarily, and the carcinogenic mechanism of ITGB2-AS1 will be further discussed in a later stage, to provide experimental basis for ITGB2-AS1 to be a potential target for early diagnosis and treatment of KIRC.
